# Building and rebuilding N-glycans in protein structure models

**DOI:** 10.1107/S2059798319003875

**Published:** 2019-04-04

**Authors:** Bart van Beusekom, Natasja Wezel, Maarten L. Hekkelman, Anastassis Perrakis, Paul Emsley, Robbie P. Joosten

**Affiliations:** a Department of Biochemistry, The Netherlands Cancer Institute, Plesmanlaan 121, 1066 CX Amsterdam, The Netherlands; b MRC Laboratory for Molecular Biology, Francis Crick Avenue, Cambridge Biomedical Campus, Cambridge CB2 0QH, England

**Keywords:** carbohydrates, crystallography, model building, *PDB-REDO*, *Coot*, N-glycans, validation

## Abstract

N-Glycosylation is one of the most common post-translational modifications and is implicated in, for example, protein folding and interaction with ligands and receptors. N-Glycosylation trees are complex structures of linked carbohydrate residues attached to asparagine residues. While carbohydrates are typically modeled in protein structures, they are often incomplete or have the wrong chemistry. Here, new tools are presented to automatically rebuild existing glycosylation trees, to extend them where possible, and to add new glycosylation trees if they are missing from the model. The method has been incorporated in the *PDB-REDO* pipeline and has been applied to build or rebuild 16 452 carbohydrate residues in 11 651 glycosylation trees in 4498 structure models, and is also available from the *PDB-REDO* web server. With better modeling of N-glycosylation, the biological function of this important modification can be better and more easily understood.

## Abstract 2

Carbohydrates are automatically built and rebuilt using *Coot* in the *PDB-REDO* pipeline.

## 1. Introduction   

Proteins are frequently regulated by post-translational modifications. One of the most common forms of such modifications is glycosylation (Zhang *et al.*, 2016 ▸); however, this is also one of the most complex forms. Glycans fulfill important roles in several biological processes, such as protein folding, stability and the recognition of other compounds (Varki & Lowe, 2010 ▸). They also greatly influence cancer progression and treatment, for instance by modifying the functionality of cell-surface receptors and adhesion molecules (Taniguchi & Kizuka, 2015 ▸).

This study focuses on the glycosylation of asparagine side chains (N-glycosylation), which is the most common form of glycosylation (Apweiler *et al.*, 1999 ▸). N-Glycosylation trees are assembled from monosaccharide moieties by glycosyltransferases and glycoside hydrolases, which handle a limited range of building blocks (Rini *et al.*, 2010 ▸). Therefore, the structures of N-glycans are predictable: they have a complex biosynthesis, but share a common five-residue core which is conserved across all eukaryotes and at least part of the Archaea (Varki & Lowe, 2010 ▸). The tree structures differ between taxa owing to their specific pathways of N-glycan processing (Rini *et al.*, 2010 ▸), and also between tissues and even between copies of a protein in the same cell. Asparagines can only be glycosylated if there is a recognition site for glycosylation. These sites, referred to as sequons, commonly have the sequence Asn-*X*-Ser/Thr, where *X* can be anything but proline (Stanley *et al.*, 2010 ▸).

**Figure 1:**
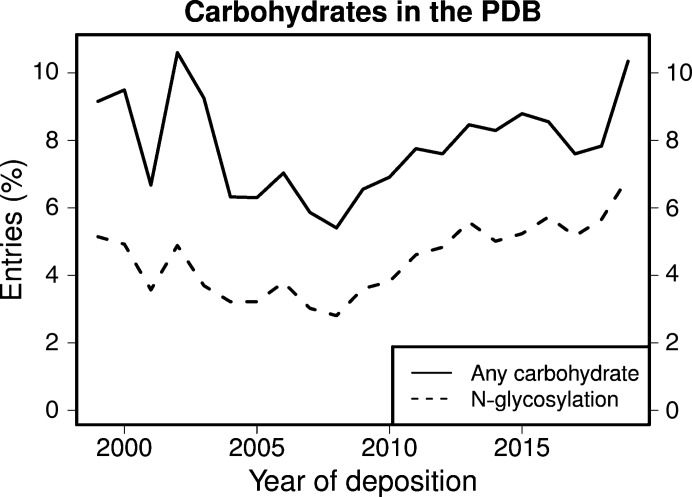
The percentage of PDB entries, per year of deposition, that contain carbohydrates or are glycosylated for the past 20 years. The percentage of carbohydrate-containing entries has been growing steadily over the last ten years.

Despite the important biological role of glycosylation, the structure quality of carbohydrates in the Protein Data Bank (PDB; wwPDB consortium, 2019 ▸) is in general inferior to protein structure quality (Crispin *et al.*, 2007 ▸). Carbohydrates exhibit many nomenclature problems (Lütteke & von der Lieth, 2004 ▸) and conformational errors (Agirre *et al.*, 2015 ▸). They are difficult to model because they are generally less well ordered, as they are typically exposed to the solvent and display high flexibility. Additionally, the median resolution of the data for glycoproteins (2.4 Å) is somewhat lower than that for PDB entries in general (2.0 Å) (van Beusekom, Lütteke *et al.*, 2018 ▸), and crystallographers are less well trained in modeling carbohydrates than protein. Also, importantly, almost all software tools for structural biology focus on the protein and deal less well with carbohydrates. Annotation at the wwPDB (Young *et al.*, 2017 ▸) deals less extensively with carbohydrates than with proteins. Recently, tools to handle carbohydrates more easily have become available, as described in, for example, Agirre *et al.* (2015 ▸) and Emsley & Crispin (2018 ▸). Although carbohydrates are frequently modeled wrongly or are not modeled at all, both the quality of carbohydrate residues and the fraction of structures in the PDB containing N-glycosylation are increasing (Fig. 1 ▸; Agirre, 2017 ▸).

*PDB-REDO* is a project that strives to improve crystallo­graphic structure models, helping crystallographers to submit better models to the PDB (Joosten *et al.*, 2014 ▸), but also makes retroactively re-refined and rebuilt models available to the user community *via* the PDB-REDO databank (van Beusekom, Touw *et al.*, 2018 ▸). Previously, improved handling of carbohydrates was introduced into the *PDB-REDO* pipeline (Joosten & Lütteke, 2016 ▸). This focused on correcting annotation issues that influenced the re-refinement process, improving some issues with carbohydrate structures. Also, it opened new opportunities for model refinement of carbo­hydrates in *PDB-REDO* (van Beusekom, Lütteke *et al.*, 2018 ▸).

Here, we describe a new software module for the *PDB-REDO* pipeline that focuses on the automated building and rebuilding of carbohydrate residues. The method uses the carbohydrate-building module recently introduced in *Coot* (Emsley & Crispin, 2018 ▸), which has been modified and extended for the purpose of this work. Three distinct operations are performed on N-glycosylation trees: poor-quality carbohydrate residues are rebuilt, existing trees are extended, and trees are added at asparagines that had not yet been modeled as glycosylated in the PDB. Also, we further improve the annotation of N-glycosylation by adding more missing LINK records between asparagine and the primary carbohydrate residue in the N-glycosylation tree, *N*-acetylglucosamine (NAG). By using these methods, the quality of carbohydrates can be greatly enhanced in a large number of existing PDB structure models.

## 2. Methods   

All methods were developed specifically for N-glycosylation, and not O-glycosylation, because this is the most prevalent and straightforward type of glycosylation and because the carbohydrate module in *Coot* (Emsley & Crispin, 2018 ▸), which is used extensively in this work, currently only deals with N-glycosylation.

The glycosylation-tree types are exactly those that were defined in the carbohydrate module of *Coot* (Emsley & Crispin, 2018 ▸): high-mannose, hybrid mammal, complex mammal, hybrid plant and complex plant. The following names are abbreviated to their PDB residue names: *N*-acetyl-β-d-glucosamine (β-d-GlcpNAc) to NAG; *N*-acetyl-α-d-glucosamine (α-d-GlcpNAc) to NDG; α-d-mannose (α-d-Man*p*) to MAN; β-d-mannose (β-d-Man*p*) to BMA; α-l-fucose (α-l-Fuc*p*) to FUC; β-l-fucose (β-l-Fuc*p*) to FUL; β-d-glucose (β-d-Glc*p*) to BGC and α-d-glucose (α-d-Glc*p*) to GLC.

### 2.1. Carbohydrate links to asparagine   

Within *PDB-REDO*, *pdb-care* (Lütteke & von der Lieth, 2004 ▸) is run to correct wrongly assigned N-glycosylation names (for example, NDG to NAG) and also to generate LINK records between asparagines and the first carbohydrate residues (Joosten & Lütteke, 2016 ▸). However, the detection of N-glycosylation in *pdb-care* is purposely conservative. Hence, glycosylation was often not detected if, for instance, the carbohydrate residue was rotated such that the C1 atom was not directly facing the asparagine. Therefore, a new program called *Carbonanza* was written which generates LINK records between asparagines and NAGs or NDGs.

For each NAG or NDG that is not linked to anything by its C1 atom, it is computed whether the C1 atom is within 6 Å of the N^δ^ atom of an asparagine. If so, the distance to the O^δ^ atom of the same asparagine is calculated: if this distance is smaller, the asparagine side chain is temporarily flipped. Next, three filters are applied: (i) the asparagine should follow the common N-glycosylation sequence Asn-*X*-Ser/Thr, where *X* is anything except proline (Stanley *et al.*, 2010 ▸); (ii) if the distance between the N^δ^ and C1 atoms is larger than 4 Å, a link will only be generated if one of the other carbohydrate atoms is within 3.5 Å of the N^δ^ atom; and (iii) to prevent the linkage of one carbohydrate residue to multiple asparagines, no link is generated if more than one asparagine fulfills all of the previous criteria. Upon generation of the LINK record, *Carbonanza* also checks whether any leaving atoms (O1 in NAG or O1L in NDG) are present: if so, these are removed.

### 2.2. Carbohydrate building and rebuilding   

#### 2.2.1. Changes to *Coot*   

Recently, the possibility of adding single residues or whole N-glycosylation trees *via* the *Coot* graphics interface was reported (Emsley & Crispin, 2018 ▸). This module allows users to add single carbohydrate residues and subsequently judge whether they are of sufficient quality to be kept, and it allows users to build entire glycosylation trees at user-defined positions. The former functionality is not used owing to the automated character of the methodology described here, while the latter is used extensively.

In the whole-tree addition, after building each residue it is decided whether this residue fits the density well enough, and it terminates automatically when all possibilities have been attempted. For the purpose of this work, the whole-tree addition method was extended such that partial N-glycosyl­ation trees can also be built. This allows the extension of existing trees in the PDB by a single residue or by multiple residues. Additionally, the carbohydrate module in *Coot* was modified such that all functionality can also be used in non­graphics mode to allow high-throughput calculations on a ‘headless’ compute server.

#### 2.2.2. Tree-type selection   

As *Coot* allows scripts to be run directly *via* the command line (Emsley *et al.*, 2010 ▸), a new *PDB-REDO* program, *Carbivore*, was written to generate a Scheme script to run the carbohydrate module in *Coot* (Emsley & Crispin, 2018 ▸). The script generated by *Carbivore* rebuilds and extends existing trees and builds new trees at previously nonglycosylated asparagines.

In its first step, *Carbivore* checks whether the protein structure model has existing N-glycosylation. *Carbivore* then determines the most suitable tree type for N-glycosylation. By default, the tree type for tree extension is set to high-mannose plus fucoses. However, if the existing tree extends beyond the five-residue core, a tree type is selected based on the residues already present: for instance, if these form a hybrid mammal tree, *Carbivore* attempts to build another hybrid mammal tree.

#### 2.2.3. Carbohydrate rebuilding and extension   

For tree rebuilding, poor-quality carbohydrate residues are cropped from the tree. Tree rebuilding then follows the same procedure as tree extension. The three-tier validation state for carbohydrates in *Privateer* (Agirre *et al.*, 2015 ▸) is used to assess the quality of carbohydrate residues in the input structure model. N-Glycans with the status ‘yes’ are of high quality and are kept; those with the status ‘check’ or ‘no’ are discarded. Additionally, all carbohydrate residues that do not fit into any of the standard glycosylation trees are deleted. Any carbo­hydrate residues further along the tree from a carbohydrate residue that was deleted are also deleted. After cropping, the tree-extension code is written to the *Coot* script for all existing trees. If a tree was fully deleted because the first carbohydrate residue was of poor quality, a whole-tree addition code is written instead.

#### 2.2.4. Whole-tree addition   

N-Glycosylation is regularly left unmodeled. Therefore, potential glycosylation sites are identified followed by tree addition (which can be regarded as extension from zero). Glycosylation sites are found using the sequon Asn-*X*-Ser/Thr. Optionally, the methodology for dealing with homologous structure models (van Beusekom, Touw *et al.*, 2018 ▸) is used to add asparagines to the list of asparagines of interest if homologous asparagines are glycosylated. This feature, however, is switched off by default, as it is only useful in the rare case of sequencing errors (see Section 3.3). Then, for each of the asparagines in the list it is checked that a chitobiose (a NAG dimer; PDB ligand code CBS) is not linked to the asparagine. If so, it is removed from the list to prevent an attempt to build a second tree at the same location. It should be noted that upon the planned remediation of the wwPDB (PDB annotators, personal communication) CBS will be replaced by two NAGs and these will be handled like all other carbohydrates automatically. Finally, code is written to try and build whole trees for each asparagine of interest.

Existing trees are first extended, followed by the attempted modeling of new trees. This decreases the risk of modeling glycosylation at the wrong asparagine when it should be modeled at another asparagine (that was already glycosylated in the input model) close by.

#### 2.2.5. Temporary deletion of carbohydrates and waters   

Before attempting to build new carbohydrate residues, some compounds that could potentially prevent correct carbo­hydrate residues from being built are temporarily removed. Firstly, chains of linked carbohydrate residues that are not linked to the protein are deleted if they are very close (<2.5 Å) to an ‘asparagine of interest’ (see above). Secondly, unlinked carbohydrate residues that are often found in N-glycosylation chains are deleted because we observed that many N-glycosyl­ation chains were poorly defined simply because the LINK records were missing. Usually, the missing LINK records lead to a distorted N-glycosylation chain because a van der Waals restraint is applied that pushes the atoms apart, instead of a distance restraint that keeps the bonded atoms together. The residue types that are allowed to be removed are limited to NAG, NDG, MAN, BMA, FUC and FUL to reduce the risk of accidentally deleting carbohydrate ligands.

All water molecules are also temporarily deleted, since they are often modeled in empty patches of density into which new carbohydrates should be modeled.

#### 2.2.6. Validation   

*Coot* is run at this point to build and rebuild carbohydrates. *Carbivore* then first determines whether there are any newly built carbohydrate residues. If so, *Privateer* is run again to assess the quality of these carbo­hydrate residues. Any newly built carbohydrate residues that are not of high quality according to *Privateer* are immediately discarded. Also, newly built carbohydrate residues are deleted if they clash strongly (<2.1 Å) with symmetry copies of existing atoms. At this point, no further checks on residue types (for example, NAG versus NDG) are required because only carbohydrate residues of the appropriate type are built by *Coot*.

Carbohydrate residues that have been built are also validated against their electron density. Note that this is already performed by *Coot* (Emsley & Crispin, 2018 ▸); however, since here glycans are added in an automated fashion, the limits for acceptance are somewhat more stringent. The new *PDB-REDO* program *stats* is run, which computes several density metrics, of which the RSCC (Jones *et al.*, 1991 ▸) and EDIAm (Meyder *et al.*, 2017 ▸) are used.

Calculating the metrics consists of several steps: recalculation of map coefficients based on the new model (for which we typically use *REFMAC*; Murshudov *et al.*, 2011 ▸), calculation of the RSCC in *EDSTATS* (Tickle, 2012 ▸), generation of an electron-density map (for example with the *CCP*4 program *FFT*; Winn *et al.*, 2011 ▸) and calculation of the EDIAm metric using the *EDIAscorer* program (Meyder *et al.*, 2017 ▸). By capturing all of these steps in a single program, *stats* speeds up the calculation and avoids additional dependencies on third-party software. *Stats* takes a structure model in mmCIF or PDB format and reflection data in mmCIF or MTZ format. Optionally, users can provide a restraint file for compounds that are not in the CCP4 dictionary (Winn *et al.*, 2011 ▸). From these data, map coefficients are calculated using the Clipper library (Cowtan, 2003 ▸). The use of anisotropic scaling and bulk-solvent correction are optional. Because *PDB-REDO* can use either X-ray or electron diffraction data, support for electron scattering factors was added to Clipper. If map coefficients are already present in the MTZ file these can also be used. The calculation of RSCC and EDIAm reimplement the published algorithms (Tickle, 2012 ▸; Meyder *et al.*, 2017 ▸), with a few modifications: the computation of the interpolated cumulative probabilities were not calculated per protein chain (as in *EDSTATS*) but by _struct_asym as defined in mmCIF space, and the electron-density radii for EDIAm computation were not tabulated as in *EDIAscorer*, but were calculated on the fly as in *EDSTATS*, dependent on the resolution and the *B* factor.

The electron-density metrics are calculated with the *B* factors of all carbohydrates set to 30.0 Å^2^. The lack of proper *B*-factor refinement is thus compensated by using a constant, relatively low *B* factor, which ensures that good density metrics are only obtained if there is electron density at decent contour levels (±1.0σ and higher in the 2*mF*_o_ − *DF*_c_ map). Empirical cutoffs were established to guarantee that few false positives will be accepted. Newly built carbohydrate residues are accepted if either the RSCC is at least 0.70 or if the sum of the RSCC and EDIAm is greater than 1.20. There is no lower boundary for the EDIAm score because we found that low EDIAm scores were often a poor indicator of carbohydrate quality; in contrast, carbohydrate residues with a good EDIAm score were indeed mostly of high quality. At a resolution of better than 3.0 Å, the density ratio that we used earlier in homology-based loop building is used (van Beusekom, Joosten *et al.*, 2018 ▸): in borderline cases, where the RSCC is between 0.60 and 0.70, if the ratio of density values between the carbohydrate residue and the main chain is at least 0.25 then the carbohydrate residue is kept. In these cases, we often observed clear electron density but with small errors in the carbohydrate modeling (causing the low RSCC) that are usually corrected by subsequent refinement. This cutoff of 0.25 was also used for loop building. It is not used at low resolution, however, because carbohydrate residues can be wrongly added in a low-resolution density blob, for instance at the end of an α-helix. For loop building, this problem was not observed: their attachment to both their N-terminus and C-terminus helps their modeling in the correct area and, compared with carbohydrate modeling, fewer candidates have to be tried because the sequence identifies exactly which loops are missing.

#### 2.2.7. Placing back carbohydrates and waters   

The remaining newly built carbohydrate residues are placed back into the model. For glycosylation trees on which rebuilding has been attempted, the old and the new versions are compared and the ‘best’ is kept. The three-tier validation by *Privateer* (Agirre *et al.*, 2015 ▸) is decisive in determining which is best: this is the tree with the largest number of carbohydrate residues that are of good quality. If this is equal, the tree with more ‘check’ statuses is kept; if this is also equal, the tree with the most carbohydrate residues is kept. If all are equal, the newly built tree is kept. If the model was refined with a flat *B*-factor model, the *B* factor of the carbohydrates is adapted to match this *B*-factor value.

Before simply deciding between the glycosylation trees before and after *Coot*, however, it is sometimes possible to generate an even better combination of the two trees. For example, if a tree of three carbohydrate residues has been deleted because the first one was poorly modeled, but only one residue has been built back, the two remaining units may be added back. This is of course only performed if the carbo­hydrates from the old tree fit the geometry of the rebuilt carbohydrate residue. Hence, the linking atoms have to be close (<2.5 Å) and the existing glycosylation chain should not clash (<2.0 Å) with the carbohydrate residues that have been restored. If this is the case, the tree is generated from parts of the old and the new glycosylation chains. If the grafted tree is kept, the necessary LINK records are also generated.

At this point, other deleted carbohydrates and water molecules can be restored. Firsly, any water molecule that does not clash (<2.5 Å) with any of the newly built residues is placed back. The same is applied to single unlinked carbohydrate residues that were deleted before. Carbohydrate chains that were unattached to the protein are restored only if none of the units in the chain clash with newly built glycans. Symmetry is always taken into account while checking clashes. Also, newly built carbohydrate residues are renumbered if there are duplicated residue numbers after restoring water molecules and other carbohydrate residues. Finally, owing to the refinement of the tree in *Coot*, the positions of the asparagine at the root of the tree and the amino acids directly before and after it are updated.

### 2.3. Implementation in *PDB-REDO*   

The new carbohydrate-handling procedures were added in *PDB-REDO* v.7.20. *Carbonanza* is run at the start of the *PDB-REDO* pipeline, just before *pdb-care* is run. *Carbivore* is run after the first refinement in *REFMAC* (Murshudov *et al.*, 2011 ▸) and all other model-rebuilding steps in the pipeline (Joosten *et al.*, 2011 ▸; van Beusekom, Joosten *et al.*, 2018 ▸), just before the second round of refinement in *REFMAC*. Carbohydrate building is switched on by default, independent of data resolution. It can be switched off from the command line if desired. Any carbohydrate residues that are added or deleted are annotated by the program *modelcompare* that writes out a visualization script for *Coot*.

### 2.4. Testing   

*Carbivore* and *Carbonanza* were executed on all PDB-REDO databank entries as of 31 October 2018. Hundreds of carbohydrates built in *Carbivore* were manually inspected to determine the optimal density cutoffs and to observe other potential shortcomings, which led to the development of the various filters in the program. Many links generated by *Carbonanza* were also checked manually, which helped to establish distance cutoffs for link generation. 2000 entries were then randomly selected from all entries in which *Carbivore* built new carbohydrate residues for optimization in *PDB-REDO*. For comparison, these entries were subjected to *PDB-REDO* once with and once without carbohydrate building. The final test set consisted of 1978 entries because some entries were not completed owing to various limitations (unrelated to carbohydrate building).

## 3. Results   

### 3.1. Carbohydrate linking   

Upon the application of *Carbonanza* to the entries present in the PDB-REDO databank, LINK records were generated for 448 NAG and 60 NDG residues in 194 entries. It should be noted that *pdb-care* (Lütteke & von der Lieth, 2004 ▸) had already been applied in the PDB-REDO databank to correct such cases. When *Carbonanza* was applied to the corresponding PDB entries, LINK records could be generated for 842 NAG and 85 NDG residues in 354 entries. The added LINK records ensure that a covalent bond is assumed by crystallographic refinement; without them, the atoms would be pushed apart by van der Waals restraints. The NDG residues that are now linked will be corrected to NAG by *PDB-REDO* using *pdb-care*.

### 3.2. Rebuilding carbohydrates   

*Carbivore* was applied to the 119 377 entries present in the PDB-REDO databank as of 31 October 2018. The current version of *Carbivore* is able to build 16 452 new carbohydrate residues in 11 651 trees in 4498 entries. Table 1 ▸ lists how many were built in each of the three separate building methods: rebuilding, extending and whole-tree addition. Also, 5818 carbohydrate residues were removed definitively: these are either poor-quality carbohydrate residues that were removed and rebuilt (note that in some cases fewer carbohydrate residues are built back than the number that were deleted) or carbohydrates that were not linked to protein and that were in the way of tree building or extension. Additionally, 6397 water molecules were removed to allow carbohydrate building.

**Figure 2:**
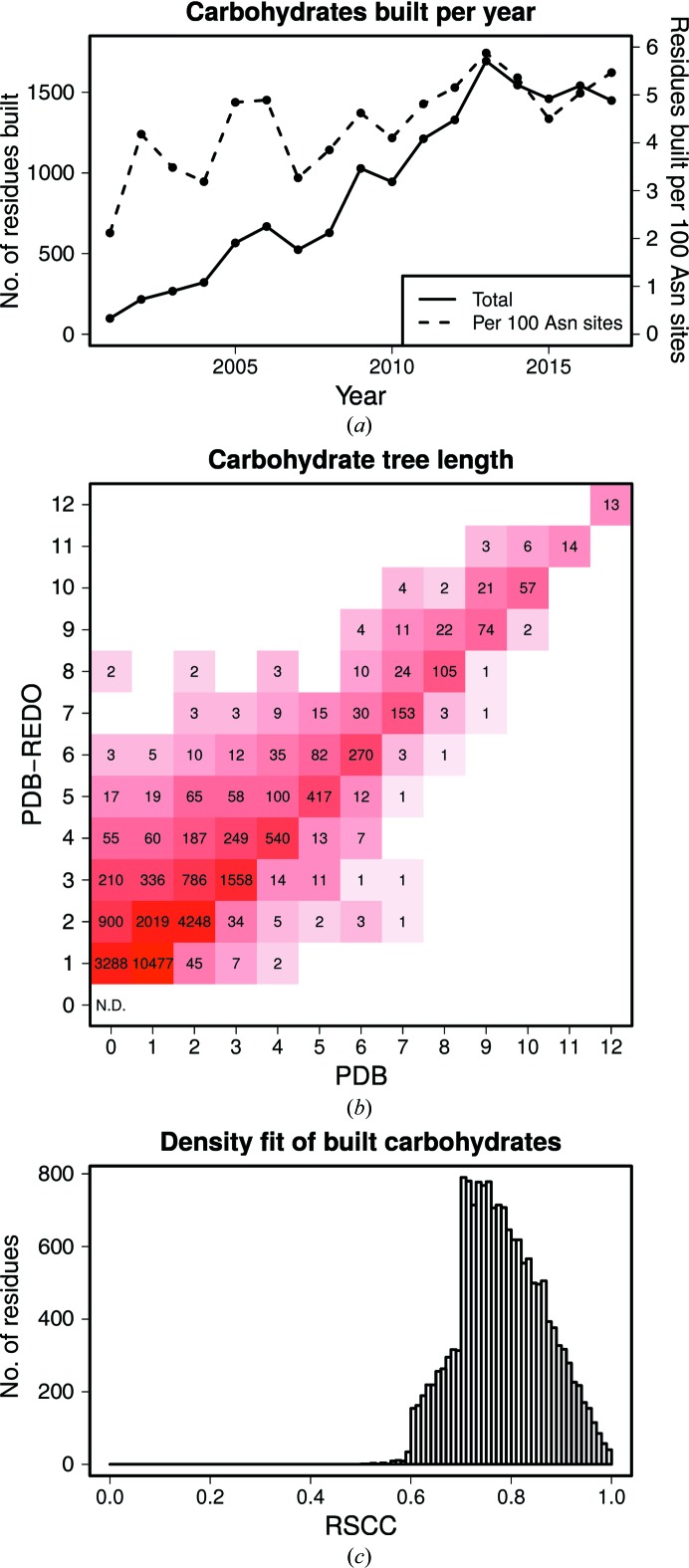
(*a*) The number of carbohydrate residues built by *Carbivore* by year of deposition in total and per 100 sequons (with sequence motif Asn-*X*-S/T). (*b*) The number of carbohydrate residues per glycosylation tree in the PDB *versus* the PDB-REDO databank. The length of most glycosylation trees remains unchanged, a considerable number of trees become longer and only a small portion of trees are shortened. The number of asparagines that are glycosylated in neither the PDB nor the PDB-REDO databank is not determined because it is not relevant here. (*c*) The distribution of the RSCC for each carbohydrate residue that was built in by *Carbivore* in current PDB-REDO entries. The sharp increases at 0.60 and 0.70 are caused by the RSCC filters (see Section 2).

The number of carbohydrate residues that can be built into the maps of deposited structure models does not depend strongly on the year of deposition: the number of carbo­hydrate residues built per 100 sequons (with sequence Asn-*X*-Ser/Thr; Fig. 2 ▸ *a*) does not change much over the course of the years; if anything, there is a slight trend that more carbo­hydrate residues have been built or rebuilt in recent years. The trend is very nearly the same if plotted against the number of amino-acid residues deposited that year. The absolute number of carbohydrate residues built increases over the years because more structure models are available.

The number of carbohydrate trees that were added is 4475; for most of these trees (3288) only the first NAG was built (Fig. 2 ▸ *b*). For the 22 291 trees that were already present, the great majority keep the same length (17 926 cases; 80.4%). A considerable minority of 4195 trees (18.8%) are extended and only 170 trees (0.8%) are shortened. The latter occurs when poorly built carbohydrate residues are deleted and replaced by fewer, but higher quality carbohydrate residues.

The density metrics for the built carbohydrates are decent: the values are good enough to allow the modeling of these carbohydrate residues but, as expected, are relatively poor compared with the values for the protein. The values of the density metrics are also an obvious consequence of the filtering performed based on these metrics. The average and median RSCC are both 0.78 (Fig. 2 ▸ *c*); these values for EDIAm are 0.38 and 0.40, respectively. The geometry of the newly built carbohydrate residues is excellent: the θ angle (which ideally should be around 0° for non-FUC residues and 180° for FUC) has average and median values of 4.8° and 1.7° for all non-FUC residues, respectively; for FUCs, the average and median are 173.3° and 175.5°, respectively. All newly built carbohydrate residues have a good geometry according to the three-tier validation state of *Privateer* (‘yes/check/no’; Agirre *et al.*, 2015 ▸).

**Figure 3:**
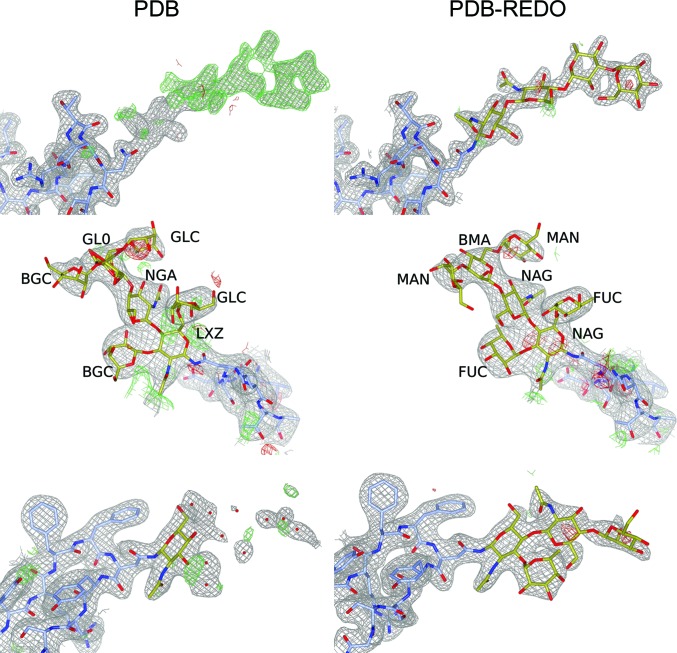
Carbohydrate remodeling: a comparison between PDB (left) and PDB-REDO (right). Top: new glycosylation-tree modeling at AsnA24 in PDB entry 2aaa (Boel *et al.*, 1990 ▸). Clear difference density is visible at this asparagine, which follows the glycosylation sequence motif. After flipping the side chain of AsnA24, four carbohydrate residues can be built at this position; there is also partial density for a fifth mannose, but this was not built. Middle: glycosylation-tree rebuilding at AsnA529 in PDB entry 3d12 (Xu *et al.*, 2008 ▸). The seven carbohydrate moieties in the PDB entry (and indicated in the figure) are carbohydrate residues that are not commonly found in N-glycosylation, which can now be replaced automatically with the correct residues. It may be possible that the wrong residue names have arisen as an unwanted side effect from PDB remediation efforts (Henrick *et al.*, 2008 ▸). The residues for which the abbreviations have not been defined (LXZ, NGA and GL0) are similar to NAG, NAG and GAL, respectively, but with one or more inverted chiral centers. Bottom: glycosylation-tree extension at AsnC81 in PDB entry 6g46 (Hussein *et al.*, 2018 ▸). Three residues could be added at this position, which was enabled partly because of improved refinement in *PDB-REDO* (*R*_free_ decreased from 23.1% to 21.5%). Ten water molecules were deleted. In all cases, amino acids are shown in blue and carbohydrate residues in gold. For sake of clarity, the 2*mF*_o_ − *DF*_c_ map is contoured at 1.2σ (top), 1.5σ (middle) and 1.0σ (bottom). The *mF*_o_ − *DF*_c_ map is shown at 3.0σ in all cases. *CCP*4*mg* (McNicholas *et al.*, 2011 ▸) was used to generate this figure.

The remodeling of carbohydrates can lead to large improvements in protein structure models. In Fig. 3 ▸, we show one example each of rebuilding, tree extension and whole-tree addition.

When *Carbivore* was run within *PDB-REDO* for 1978 entries (randomly selected from the 4498 entries in which carbohydrates could be built), it built 7001 carbo­hydrate residues. 2869 poorly built carbohydrate residues were also deleted, as well as 2562 water molecules. Most carbohydrate residues (50) were built in PDB entry 4ubd (Wu *et al.*, 2015 ▸), while 22 residues were deleted in this entry to allow rebuilding; thus, a net gain of 28 carbohydrate residues was achieved. As expected, the impact of carbohydrate building on the overall performance of *PDB-REDO* is minimal. On average, the *R*_work_ and *R*_free_ increase by negligible amounts (1.2 × 10^−5^ and 2.6 × 10^−4^, respectively). The geometrical scores change even less: the Ramachandran *Z* score and the first-generation packing *Z* score, both computed by *WHAT_CHECK* (Hooft *et al.*, 1996 ▸), decrease by 0.008 and 0.003, respectively.

It was observed that the *R*_free_ deteriorated in some cases where a flat *B*-factor model was applied in refinement. The modeled carbohydrates are clearly supported by electron-density evidence, although negative electron difference density appears. This is owing to the carbohydrate residues having greater mobility than the protein average, yet they are modeled with the same *B* factor. This is a clear drawback of the flat *B*-factor model that is applied to reduce the overall number of model parameters. These cases may therefore be improved by using alternative, low-parameter *B*-factor models where at least the *B* factors of the carbohydrate residues are separated from those of the protein. Without taking all flat *B*-factor entries into account, there is a (tiny) overall improvement in *R*_free_ of 1.9 × 10^−4^ instead of a deterioration of 2.6 × 10^−4^.

We have not ‘redone’ all structure models in which carbohydrates can be built yet owing to computational constraints. The entries that were not part of the test set will be renewed gradually.

### 3.3. Whole-tree addition   

Based on the methodology that we previously developed to map homologous structure models onto one another (van Beusekom, Touw *et al.*, 2018 ▸), we added the option to try to build trees if homologous asparagines are glycosylated. However, we disabled this functionality by default because the computational cost outweighs the value of the results. In total, only 35 trees were built that could not be built based on the sequence. Of these trees, ten cases were built near gaps in the protein model: the Asn-*X*-Ser/Thr sequence criterion was not fulfilled because either the *X* or the Ser/Thr was disordered. In such cases, it is better not to build trees because the electron density is usually relatively poor and carbohydrates were sometimes built into the density of the missing main chain. In 14 cases, the built carbohydrate residues seemed correct but the sequence may be wrong; 10 of these 14 cases were found in PDB entry 3red (C. B. C. Cielo, T. Yamane, Y. Asano, N. Watanabe, A. Suzuki & Y. Fukuta, unpublished work) at Asn118 in different chains. The local sequence, Asn-Thr-Lys, does not fulfill the Asn-*X*-Ser/Thr sequence motif. However, HSSP alignment (Touw *et al.*, 2015 ▸) shows that in the gene associated with the PDB entry and in the closest homologs there is a serine at the position of the lysine. The protein-sequencing procedure is not detailed, but the gene associated with the PDB entry is only 89% identical and the protein was isolated from a natural source. Hence, we cannot conclude from the sequence data whether this is an error or not, but the lack of density for the side chain of Lys120 suggests that it may indeed be a sequence error. Finally, the remaining 11 cases in which carbohydrate residues were built on homology were probably or unequivocally wrong. Either the density was uninterpretable, the density was not carbohydrate density or there was density from another glycosylated asparagine nearby into which the carbohydrate residues were modeled.

## 4. Discussion   

Building carbohydrates in protein structure models has been difficult, in part owing to a lack of suitable computational tools. Therefore, the structural quality of carbohydrates has traditionally been poor. Recently, much improved tools for carbohydrate building have become available. This motivated us to apply them to existing protein structure models to make improved structure models available for glycosylated protein structures determined in the past and to simultaneously make these tools available to crystallo­graphers *via* an automated web server.

**Figure 4:**
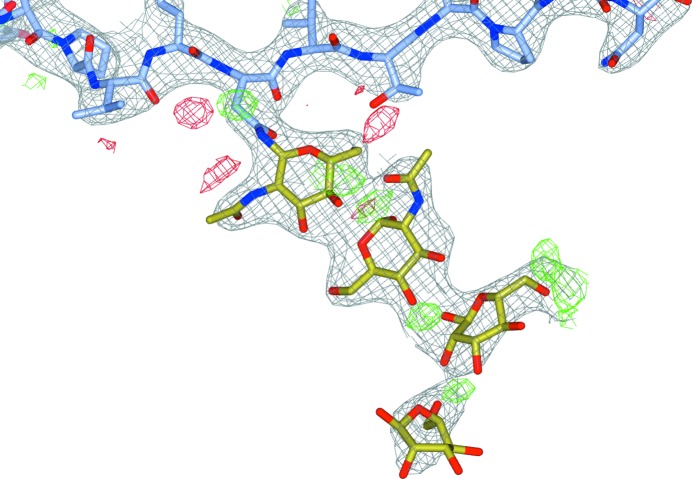
Glycosylation tree at AsnG165 in PDB entry 1mql. The LINK records between the different carbohydrate residues are missing, causing the carbohydrate residues to be pushed apart owing to van der Waals restraints. This is exacerbated by the leaving ‘O1’ atoms that were not removed when the carbohydrate tree was built. The 2*mF*_o_ − *DF*_c_ map and the *mF*_o_ − *DF*_c_ map are contoured at 1.5σ and 3.0σ, respectively. *CCP*4*mg* (McNicholas *et al.*, 2011 ▸) was used to generate this figure.

Previously, we have dealt with the annotation issues of glycosylated protein models, as this is crucial for carbohydrate refinement and subsequently for building N-glycosylation trees. Here, we first present an improved methodology to improve the linking of NAGs and (wrongly named) NDGs to asparagine. Generating these links goes beyond improvement in annotation, as the refinement of the subsequent model is improved because the covalent bond is now taken into account. At present, we have only dealt with links from asparagine to the first carbohydrate residue in the glycosylation tree. This may be extended to also generate links between two carbohydrate residues further in the chain, which would potentially improve the refinement of glycosylation trees such as in PDB entry 1mql (Ha *et al.*, 2003 ▸; Fig. 4 ▸). However, automatically generating such links leads to new problems since unlinked carbohydrate residues are often too far away from one another. If multiple LINK records are then generated to pull them together, this leads to incorrect conformations and to bonds that are too long. Therefore, we chose instead to attempt to rebuild such carbohydrate residues by first removing them and then extending the tree.

We show here that in many cases carbohydrates may be built fully automatically. However, for multiple reasons, N-glycosylation trees are difficult to build to completion. First and foremost, the electron-density quality rapidly falls along the glycosylation chain as disorder increases away form the protein surface. This often makes it difficult to decide whether a carbohydrate residue is ‘good enough’, especially in an automated fashion. Additionally, carbohydrate residues can only be built well if the previous units in the tree are properly modeled. If a single unit is not built in the optimal position, building the next one is likely to be unsuccessful. Moreover, the six-membered ring is symmetrical enough to sometimes be wrongly flipped 180°, despite the presence of (small) chemical groups on the ring. It therefore remains important that crystallographers manually inspect, and if necessary modify, automatically built glycans: they cannot (yet) be automatically built as well as protein.

Since this work depends on the carbohydrate-building module in *Coot*, it also has similar limitations as those discussed in Emsley & Crispin (2018 ▸): glycosylation trees are not always built to completion (especially at lower resolution), temperature factors are crudely estimated, which impacts the density-fit calculations, and the range of glycosylation trees that may be built is limited. In the current work, the available range is even much more limited, since no ‘expert user mode’ is available to add any carbohydrate residues outside of the five standard trees defined within the method. The *B*-factor estimates could be improved by a short refinement (as we have performed previously for loop modeling; van Beusekom, Joosten *et al.*, 2018 ▸) or by using shift-field maps (Cowtan & Agirre, 2018 ▸); however, the computational costs currently outweigh the merits of better *B*-factor estimation. The carbohydrate building here is also more conservative than in *Coot*, since we apply additional filtering steps. This leads to fewer cases where ‘probably (but not unequivocally) wrong’ (Emsley & Crispin, 2018 ▸) carbohydrate residues are built, but also further increases the limitation that glycosylation trees are not built to completion. In the context of *PDB-REDO*, carbohydrate remodeling is followed by reciprocal-space refinement, which may lead to more interpretable maps that allow users to attempt to further extend carbohydrate trees, both manually and automatically, in subsequent model-improvement rounds.

In earlier work (van Beusekom, Lütteke *et al.*, 2018 ▸), we showed that *Privateer* labeled over 10 000 carbohydrate residues as wrong or doubtful both in the PDB and in the PDB-REDO databank. Since that analysis, the number of entries in the PDB-REDO databank has increased by 10 266 entries (9.2%). Hence, the expected number of problematic carbohydrate residues is now above 11 000. In this paper, we show that we can rebuild 6364 carbohydrate residues: this accounts for about half of the problematic residues. The other problematic carbohydrate residues cannot be rebuilt automatically, for instance because of a lack of electron-density evidence or current limitations to our methods. This illustrates again that automated carbohydrate building can solve many, but not all, problems.

We observed that small changes in the coordinates and in the electron density can lead to different numbers of carbohydrate residues being built. Unrelated changes in the *PDB-REDO* pipeline, such as an update of the *REFMAC*5 program (Murshudov *et al.*, 2011 ▸), can lead to different results, because the coordinate file and the map coefficients submitted to *Carbivore* are slightly different. Sometimes this leads to better results and sometimes the results deteriorate; averaged over many entries, these are minor differences. As stated before, manual analysis is thus still required for optimal results.

A potential improvement to our methods is better selection of which carbohydrate tree type is built. Currently, by default, we attempt to build a mannose tree with two FUCs on the first NAG: a tree that is not found in nature. Building of such an unnatural tree currently happens in one case (the glycosyl­ation tree at AsnA638 in PDB entry 4p44; Novakova *et al.*, 2016 ▸). However, residues are rarely built past the common core of five residues equivalent in all glycosylation trees (Fig. 2 ▸ *b*). To circumvent cases like this as much as possible, we revert to building other tree types if other glycosylation trees in the structure model extend past the common core: this type then becomes the most likely candidate. For example, in PDB entry 5fji (Agirre *et al.*, 2016 ▸) high-mannose trees are found: we therefore attempted to build more high-mannose trees. However, this tree type is also not necessarily correct since different glycosylation trees can be found within a single protein molecule [for example in PDB entry 5t3x (Gristick *et al.*, 2016 ▸), where we find high-mannose trees but also trees that are complex plant or complex mammal]. It is even possible that different glycosylation states are found in protein copies within the same crystal. Hence, it is possible that incorrect carbohydrate residues are sometimes built. Also, there are likely to be a few carbohydrate residues that were not built because an attempt was made to build the wrong tree type, and building the wrong carbohydrate residue type into the density of another is likely to be less successful.

To improve choosing the correct tree type to build, outside annotation could be helpful. For instance, if a protein is expressed in a human cell line it should not contain plant glycosylation trees. In principle, the PDB file contains information about the source organism in which the protein was expressed. However, we do not find this annotation to be very reliable: for example, a large fraction of N-glycosylation trees are found in entries that were supposedly expressed in *Escherichia coli*. While glycosylation exists in Gram-negative bacteria (Benz & Schmidt, 2002 ▸), it is not very common and such a large number of entries would not be expected. However, the annotation has been improved over the years (Henrick *et al.*, 2008 ▸; Sen *et al.*, 2014 ▸), so the use of species information may be possible for more recent entries.

The methodology presented here is another step in the automated handling of carbohydrate moieties in crystallo­graphic methodology. Instead of only identifying the issues, they can now be corrected by automated rebuilding. Additionally, carbohydrate residues are automatically built to extend existing glycosylation trees and to add glycosylation trees where they were missing. Although we still recommend manual checking, building correct N-glycosylation trees has become much easier.

## 5. Availability   

Both the PDB-REDO databank and web server are hosted at https://pdb-redo.eu. Crystallographers can submit work-in-progress models on the web server to run *PDB-REDO* including the carbohydrate-building procedure. The 1978 models from the test set are available through the databank. Other databank entries will be updated gradually to include the carbohydrate-building procedure. On the PDB-REDO databank entry pages, registered users can submit an update request to prioritize the update of that entry. Binary executables for *Carbivore*, *Carbonanza* and *stats* are available from the PDB-REDO website and the source code is available on request. Non-graphics-dependent carbohydrate modeling in *Coot* has been available since June 2018.

## Figures and Tables

**Table 1:** Number of carbohydrate residues built in PDB-REDO entries available as of 31 October 2018

Process	No. of residues built	No. of entries modified
Rebuilding†	6364	1961
Tree extension	4031	1721
Whole-tree addition	6057	2372
**Total**	**16452**	**4498**

† This also includes carbohydrate residues that were built when a rebuilt tree could be further extended.
